# Supplementary material for: Are carbon emissions trading and green financial instruments synergistic? -Comprehensive quantitative research based on content analysis
Source: PLoS One. 2024 Mar 7;19(3):e0298601. doi: 10.1371/journal.pone.0298601 (PMC10919601; doi:10.1371/journal.pone.0298601)
Supplement: S1 Data — (ZIP) [file pone.0298601.s001.zip › Data/地级市Z_F工作报告.zip!/░▓╗╒/│╪╓▌╩╨/└┤╫╘╣1⁄2╓┌║┼í╛┬φ┐╦╩2╛▌═°í┐_2019.docx]

各位代表：

现在，我代表市人民政府，向大会报告工作，请予审议，并请市政协委员和其他列席人员提出意见。

**一、2018年工作回顾**

刚刚过去的2018年，是极具挑战、极不寻常的一年。面对一系列新困难、新问题、新考验，全市人民在省委、省政府和市委的坚强领导下，以习近平新时代中国特色社会主义思想为指导，坚定贯彻新发展理念，深入实施五大发展行动计划，凝心聚力破难题，扑下身子抓落实，经济运行总体平稳、稳中有进，市四届人大一次会议确定的主要目标任务较好完成，高质量发展迈出新的坚实步伐。预计，全市生产总值680亿元、增长5%左右，财政收入107.2亿元、增长5%，固定资产投资增长10.5%，社会消费品零售总额增长10.5%，城乡居民人均可支配收入分别增长8.1%、9%。一年来，主要做了以下工作。

**（一）落实“六稳”政策措施，经济保持稳定增长。**制定促进经济高质量发展、民营经济发展等系列政策。着力稳投资，扎实开展项目建设年活动，长九一期、中建材“三新”产业园3D手机盖板、德斯兰卡年产5万吨铝基型材、华尔泰年产30万吨双氧水、振宇汽车零部件等98个亿元以上项目竣工投产，阿李新能源产业园、得奇环保表面处理中心、泰富安东智能制造、童车产业园等224个项目开工建设，569个市重点项目完成投资480亿元、增长12.4%，工业投资增长20%以上，民间投资增长18%。着力稳金融，创新推出易旅贷、政融保业务，小微企业获贷户数增加1800户，化解不良贷款9.9亿元，新增信贷投放53亿元；远航港口港股上市，艾可蓝报会在审，新增“新三板”、四板挂牌企业11家，运用产业投资基金孵化企业50家。着力稳外贸，在首届中国国际进口博览会上成功签订4.5亿美元进口大单，对“一带一路”沿线国家出口8200万美元、增长10%，进出口总额7.7亿美元，第4次跻身“中国外贸百强城市”。着力稳外资，新设立外资企业7家，实际利用外资3.93亿美元。着力稳就业，城镇新增就业2.1万人、完成省定目标的150%，农村转移劳动力1.8万人，实现城镇零就业家庭动态“清零”。着力稳预期，常态化开展“四送一服”双千工程，实施“千干帮千企”活动，兑现市级政策奖补资金1.2亿元，减税降费减轻企业负担15.95亿元。规模以上工业增加值增长8.5%、利润增长50%，税收超千万元企业突破100家，贵航特钢首次进入中国民营企业制造业500强。

**（二）加快新旧动能转换，质量效益明显提升。**编制实施战略性新兴产业发展规划，省级半导体战新基地实现产值54亿元、增长10%，新增省重大专项1个，世界最薄6微米电子铜箔实现量产，战略性新兴产业产值增长23%。实施工业企业技改升级示范项目100个、竣工投产50个，新增国家级和省级绿色工厂6家、省级专精特新企业20家。引进高层次创新创业人才团队3个，入选省第四批“特支计划”领军人才6人、居全省第3，池州高新区申创国家级高新区进入科技部初审，荣获省科技进步二等奖项目1个，净增高新技术企业25家、为历年最高，高新技术产业增加值增长17%以上。加快全域旅游示范区创建，九华山创建世界地质公园通过联合国教科文组织专家评估，牯牛降创建5A级景区完成规划编制，天下粮仓文旅园等10个亿元以上项目建成开放，新增4A级旅游景区1家，接待游客量、旅游总收入分别增长10%、12%。在全省率先出台《养老机构医养结合服务规范》，朱备禅修小镇入围全国最美特色小镇50强，九华山森林特色小镇入选首批国家试点，成功举办池州国际马拉松、2018游学养大会。新增省级服务业集聚区1个、省级特色小镇2个。快递业务量突破1000万件。完成农业“两区”划定，建成高标准农田12.2万亩，粮食总产66万吨；新增省示范农业产业化联合体8家，新增国家生态原产地保护产品5个、总数居全省第1；农产品网络销售额15.9亿元，池州国家农业科技园区进入“国家队”，石台县入选全国农村一二三产业融合发展先导区。

**（三）聚焦污染防治攻坚，环境质量显著改善。**出台主体功能区规划，生态保护红线面积比重达到33.5%。制定实施水清岸绿产业优美丽长江经济带（池州段）“1+7+1”方案，整治“散乱污”企业332户、入河排污口58处，完成畜禽养殖禁养区、限养区调整划定。强力推进中央、省环保督察等环境突出问题整改，开展万名干部群众大排查大整治行动，原前江工业园区违规堆放工业固废全面整治，升金湖核心区湖面实现“三无”、缓冲区违建全部拆除，东至经开区河沟水质稳定达标、安全防护距离内居民全部搬迁，贵铜公路沿线鸭棚养殖污染彻底根治，主城区滨江片区环境沉疴实现出清。创建绿色矿山14家、整顿关闭9家；拆解非法采砂船舶79艘，成功打掉涉案35人的跨两省六市非法盗采江砂团伙，非法砂场基本清除，主城区和县区砂石集散中心开工建设。PM_2.5_、PM_10_平均浓度降幅和空气优良天数比例增幅均居全省前列。秸秆禁烧监管平台基本建成，地表水断面、空气质量生态补偿机制建立运行，工业固体废物、医疗废物得到有效监管。单位规模工业增加值能耗下降4.5%以上。东至、青阳经开区获批省级循环化改造园区。河湖长制纵深推进，建成全省首批水生态文明城市。林长制体系全面建立，造林绿化3.6万亩。

**（四）强化基础设施建设，城乡融合步伐加快。**市县空间规划体系初步建立。海绵城市试点任务全面完成，六大片区城市海绵体全面构建。东部产城融合创新区开工建设。建成城市绿道、“白改黑”道路20公里，新增城市绿化面积66.7万平方米，天堂湖公园建成开放，环平天湖“花海”成为一道靓丽的城市风景。扎实开展“两治三改”专项行动，改造老旧小区12个，治理城乡违法建设47万平方米。城市公交体制改革基本完成，新的城市公交集团正式运营，新增新能源公交车48台。新一轮文明城市创建扎实推进。生活垃圾分类试点有序实施，大中型餐饮企业餐厨垃圾集中处置实现全覆盖。编制实施乡村振兴战略规划，建成美丽乡村省级中心村43个；大力推进农村环境“三大革命”，35个乡镇政府驻地、107个中心村污水处理设施基本建成，农村危房改造3144户、农村改厕2.6万户，三县一区城乡环卫一体化落地运行，4G网络、公交通达、直接通邮实现建制村全覆盖。池州长江公路大桥主塔封顶，池黄高铁、G3W德上高速池州段、九华山机场扩建和G318池州至殷汇段等3条国省干线一级公路开工建设，农村道路畅通工程圆满收官，国省道大中修210公里。长江干流单体规模最大的长九码头一期、江口港区三期和秋浦河航道整治工程开工建设，青通河航道整治疏浚9公里，建成二类渡口“渡改桥”工程2个。平天湖二站等4处主城区除涝骨干工程、秋浦河等7处中小河流治理、20座小水库除险加固工程全面完工，解决4.7万农村人口饮水安全问题。

**（五）持续深化改革开放，发展活力不断增强。**推行“证照分离”改革，全面实行“双随机一公开”监管，新增私营企业4645户、个体工商户1.2万户，分别增长14.7%、10.3%。“一网一门一次”改革取得突破，市级网上可办事项达99.8%，企业开办、不动产登记、工业建设项目施工许可证办理时间分别压缩至3个、5个和45个工作日以内。税收征管体制、城市综合执法、盐业体制等改革基本完成。国资国企改革全面推进，九华山文旅集团组建运营，完成12家市属经营类事业单位转企改制。在全省率先设立PPP引导基金，2个PPP项目入选财政部示范项目案例。全面完成存量政府债务置换，清理盘活存量资金6.1亿元，财政收入中非税占比下降6.6个百分点。市直单位公有住房清理基本完成。整市推进农村集体产权制度改革，集体资产清产核资工作基本完成，“三变”改革试点实现涉农乡镇全覆盖，东至县入选国家级农村综合性改革试点。完成池州高新区与前江工业园区优化整合，大渡口经开区（石台经开区）体制机制进一步理顺。招商引资提质增效，省外亿元以上项目到位资金450亿元，其中15亿元以上项目15个、比上年增加6个。与国际友城合作交流领域不断拓展，池州的“朋友圈”进一步扩大。

**（六）致力改善民生福祉，社会事业全面进步。**33项民生工程全面完成。启动打赢脱贫攻坚战三年行动，大力实施脱贫攻坚“十大工程”，投入扶贫资金4.1亿元，市本级先行安排1亿元支持石台县脱贫摘帽，产业项目到村、到户覆盖率分别达到100%、97.1%，易地扶贫搬迁工程全面完成，全市贫困村全部出列，2.04万贫困人口脱贫。基本完成机关事业单位养老保险制度改革，城乡居民基本养老保险最低标准每人每年提高420元。城乡低保标准分别提高6%、8%，老年人“三项补贴”、残疾人“两项补贴”全面实施。建成45个三级居家养老服务中心。新改建公办幼儿园9所、主城区中小学校7所，“全面改薄”圆满收官，基本消除66人以上超大班额。组建池州职业教育集团。综合医改扎实推进，建成紧密型医共体5个。全面两孩政策稳步实施。举办安徽（池州）地域文化论坛，文物大普查大保护新增文物点205处，评选首届文化遗产守望者10人，2镇1村入选第七批中国历史文化名镇名村。12人入列“中国好人榜”“安徽好人榜”。民族、宗教、妇女、儿童、老龄、残疾人、红十字等事业不断发展，对台、侨务、供销、地震、档案、保密、地方志、新闻出版、社会科学、援疆援藏等工作取得新成绩。

圆满完成村居委换届。社会治安防控体系不断完善，扫黑除恶专项斗争深入开展，打掉非法控制招投标、砂石、混凝土市场等犯罪团伙61个，人民群众安全感指数居全省前列。建成信用信息共享服务平台，惩戒失信者2615人。安全生产、食品药品安全、信访形势持续稳定。建成气象防灾减灾中心、人防基本指挥所。军民融合深度发展，国家安全、国防教育、国防动员和双拥优抚安置工作持续加强。池州烈士陵园被评为省级爱国主义教育基地。

坚持把政治建设摆在首位，扎实开展“讲忠诚、严纪律、立政德”专题警示教育，政府自身建设全面加强。完善重大事项合法性审查、政府法律顾问、公众代表列席政府常务会议制度。自觉接受人大监督，依法执行人大决议决定，办理人大代表议案、建议135件。主动接受政协民主监督，开展各类民主协商10项，办理政协委员提案349件。严格落实党风廉政建设主体责任，加强巡视巡察反馈问题整改，实现审计监督范围、对象全覆盖，扎实开展机关作风效能集中整顿，政务环境持续改善。

各位代表！回首2018年，我们迎接了中央生态环保督察“回头看”、省环保督察，经历了突出环境问题集中整改、佛教商业化治理、九华山风景名胜区违法建设整治、非洲猪瘟防控等一系列急事难事，承受了前所未有的考验。这些长期以来发展中积累的矛盾和问题集中凸显、相互交织，是实现高质量发展必须跨越的重大关口。我们在市委的坚强领导下，以壮士断腕、刮骨疗毒的决心，化挑战为机遇，举全市之力坚决攻克了一批难啃的硬骨头，生态优先、绿色发展的理念成为全市上下的思想共识和共同行动，各级干部在贯穿全年的整改攻坚中经受了历练、提升了站位、锤炼了作风、增强了本领。实践昭示我们，只有将新发展理念入脑入心，才能保持定力、直面挑战、敢于担当，才能善作善成、少走弯路、闯出新路。只要我们坚持新时代新担当新作为，锐意进取，砥砺前行，就一定能开创现代化“三优池州”的美好未来！

各位代表！一年的奋斗充满艰辛，取得的成绩殊为不易。这是省委、省政府坚强领导和关心支持的结果，是市委团结带领全市人民勠力同心、扎实苦干的结果，是市人大、市政协和社会各界监督支持的结果。在此，我代表市人民政府，向在各个岗位上辛勤工作的全市人民，向给予政府工作大力支持的人大代表和政协委员，向各民主党派、工商联、各人民团体和社会各界人士，向驻池解放军指战员、武警官兵、政法公安干警和消防救援队伍指战员，表示崇高的敬意！向关心支持池州改革发展的中央和省驻池单位，向在我市创业的投资者、建设者，表示衷心的感谢！

去年是改革开放40周年，也是池州复建而立之年、撤地建市成人之年。我们举办纪念改革开放40周年暨池州复建30周年成果展等系列活动，见证改革开放波澜壮阔的历史巨变，感受新池州由小到大、筚路蓝缕的奋进历程，重温艰苦创业、负重拼搏、开明开放、务实创新的池州精神。站在新的历史起点，我们没有任何理由自我满足，没有任何理由止步不前，必须干字当头，接力奋斗，继续高举发展的大旗，以永不懈怠的精神状态和一往无前的奋斗姿态，走好新的“长征路”，闯出发展新天地。

我们也清醒地认识到，发展不平衡不充分的问题依然突出，工作中困难和短板仍然不少，池州的优势和潜力尚未充分释放，主要表现在：实体经济发展面临诸多困难，重大产业项目支撑作用没有完全显现，经济发展内生动力尚显不足，生产总值指标受核算支撑因素影响没有达到年初预期，教育、医疗、文化、养老、生态等民生欠账较多，影响社会稳定的突出矛盾还没有彻底解决；关键在于经济发展还没有完全摆脱传统路径依赖，能耗强度、总量“双控”压力较大，创新要素集聚不明显，新兴产业拉动能力不足；根子在于作风、效能、能力建设亟待加强，少数干部事业心责任心有待提升，研究运用政策和依法行政的水平不高，优化发展环境还需要付出艰苦的努力。我们要正视矛盾和困难，采取更加有力的措施，认真加以解决。

**二、2019年工作安排**

今年是新中国成立70周年，是全面建成小康社会关键之年。前进道路上，我们面临着要素资源吸附力不足、新旧动能转换不畅、发展压力增大等艰巨挑战，诸多短板制约还将显现，必须做好应对更加复杂严峻挑战的准备。同时更要看到，我市经济发展健康稳定的基本面没有变，经过三大攻坚战的洗礼，各类风险隐患逐步化解，结构调整阵痛期正在加速迈过，经济增长有望步入轻装上阵的轨道，仍然处在大有可为的战略机遇期。我们要抓住国家新一轮改革开放、长三角区域一体化发展上升为国家战略等新战略新机遇，贯彻新理念，提升新坐标，拓展新视野，坚定信心，把握主动，奋力开创新时代高质量发展新局面。

今年政府工作的总体要求是：以习近平新时代中国特色社会主义思想为指导，全面贯彻党的十九大、十九届二中、三中全会精神和中央、省委经济工作会议精神，按照市第四次党代会和市委四届八次全会部署，坚持稳中求进工作总基调，坚持新发展理念，坚持推动高质量发展，坚持以供给侧结构性改革为主线，坚持深化市场化改革、扩大高水平开放，继续打好三大攻坚战，统筹推进稳增长、促改革、调结构、惠民生、防风险工作，进一步稳就业、稳金融、稳外贸、稳外资、稳投资、稳预期，保持经济持续健康发展和社会大局稳定，为全面建成小康社会收官打下决定性基础。

综合考虑，今年经济社会发展的主要预期目标是：地区生产总值增长6.5%左右，财政收入增长6%，规模工业增加值增长8%左右，固定资产投资增长10%以上，社会消费品零售总额增长10%，进出口总额7.7亿美元以上，游客接待量增长10%以上，旅游总收入增长12%以上，城乡居民收入分别增长8%、8.5%，居民消费价格涨幅3%左右，城镇新增就业1.4万人、调查失业率控制在5.5%以内，节能减排等约束性指标完成省定目标任务。

为此，要重点抓好以下六个方面工作：

**（一）突出稳投资强实体，着力筑牢经济高质量发展根基**

更大力度推进项目建设。全年新引进省外亿元以上项目150个以上、到位资金470亿元，确保新开工亿元以上重点项目150个以上，建成80个以上。坚持和完善分级分层精准调度机制，以季度为单元组织项目开竣工观摩测评。突出基础设施、生态环保、乡村振兴等重点领域补短板，当年投资80亿元以上。遴选10个重大项目分别成立工作专班衔接推进，确保列入国家和省专项规划、投资计划达到应有份额。强力推进招商引资落地见效，组建产业发展研究专班，跟踪研究、定期梳理发布主导产业招商项目和招商行动路线图，加强与央企、知名民企、外企合作，综合采取资源整合、股权合作、基金运作等产融结合招商新模式，落实招商项目全程代办制，签约项目转化率60%以上。

推进开发区高质量发展。加快开发区“去行政化”，创新管理体制、运营机制和绩效考核体系，将70%以上的人员、70%以上的精力用于招商引资和项目推进。分级分类制定授权清单，依法下放经济管理权限，推进“一枚公章管审批”。严把投资强度、产出效益、环保安全等关口，推进省级以上开发区环保安全达标改造。建立主城区东部三个园区协同发展联动机制。加快江南产业集中区集中连片布局、节约集约用地，更大力度承接产业集群转移，税收增长20%以上；市开发区加大半导体基地建设和税源型企业培引力度，税收千万元企业突破20家。支持池州高新区以创建国家高新区为契机，加快特色产业培育和前江片区材料基地建设；东至经开区坚决守住环保安全底线，推动落户项目尽快开工；大渡口经开区（石台经开区）合作共建落地见效；青阳经开区在引进大项目上取得突破，走出一条特色化、差异化的高质量发展路径。

促进民营经济大发展。设立1亿元民营经济专项资金、5亿元中小企业产业发展基金、2亿元民营企业纾困救助基金。严格落实减税降费政策，抓好供给侧结构性改革降成本行动，确保企业社保缴费负担总体上有实质性下降。落实无还本续贷政策，扩大新型政银担、税融通、劝耕贷、续贷过桥周转贷款等业务规模，小微企业贷款增长10%以上，金融机构贷款增幅达到全省平均水平。推进艾可蓝首发上市、安芯电子报会，力争新增上市辅导企业2家以上，“新三板”、四板挂牌企业10家以上。开展民营企业大走访，帮助解决转型、融资、用工、市场开拓等难题。建立民营企业投诉维权中心。继续实施企业家轮训工程，开展优秀民营企业和企业家评选表彰，全力支持民营企业走向更加广阔的舞台。

**（二）突出调结构促升级，着力加快“4+3”现代产业体系建设**

推动制造业向中高端迈进。建立分产业推进机制，完成工业投资190亿元，继续实施百户企业技改示范项目，新增规模工业企业60户以上。现代装备制造产业，重点加快飞渡重工、智能拉链装备产业园、泰富安东智能制造、童车产业园等40个亿元以上项目建设，确保丁是丁精密零部件、柯力传感器三期等15个项目建成投产。现代化工产业，重点加快红太阳生命产业园、新北卡小分子靶向医药、泰合森年产50万吨清洁汽油、广信离子膜烧碱等30个亿元以上项目建设，确保三禾化工等15个项目建成投产。金属非金属材料产业，重点加快长九二期、西恩锂电池回收及综合利用、贵航100万吨钢生产线升级改造、安安科技铝基材料基地、阿李新能源产业园等60个亿元以上项目建设，确保长智PC构件一期等10个项目建成投产，启动1—2条矿产品专用运输廊道规划建设。加快战略性新兴产业发展，重点加快铜冠铜箔三期、华芯电子6英寸芯片、微半半导体分立器件芯片、深兰科技人工智能、云计算中心及大数据产业园等25个亿元以上项目建设，确保华宇创芯封测产业园、赛米科电子、红利得光电等10个项目建成投产，省级半导体基地增速快于全省平均水平。加快大院大所合作对接成果转化，发挥科技创新券等科技促进政策作用，新引进3个左右高层次人才团队，高新技术企业突破100家。

提高全域旅游供给质量。深入推进国家全域旅游示范区创建、国家级旅游业改革创新先行区建设。完成九华山、齐山—平天湖风景名胜区总体规划修编。推进九华山旅游业态向休闲度假、健康养生、文化研学、山地运动转型，支持牯牛降5A级景区、九华山世界地质公园创建。启动平天湖山居小镇项目，丰富杏花村文化旅游区业态功能，加快东九华、礼貌山等休闲度假区产品开发，大力发展精品民宿。制定乡村旅游三年行动方案，选择10个建制村或较大自然村创建乡村旅游示范村。建成市旅游集散中心，启动1条“交通+旅游+驿站”最美风景道建设。发挥文旅集团投融资和文旅产业基金引导孵化功能，加快全市旅游资源整合和联投联销。完成旅游综合执法改革。建立过夜游客统计制度。

壮大体育健康养生等现代服务业。设立大健康产业发展办公室，完善产业支持政策。加快石台富硒氧吧小镇等4个省级特色小镇建设，推动杏花村智慧康疗小镇、傩戏小镇、宝藤医疗大数据产业园、莲花山颐养小镇开工建设。扩大池州国际马拉松等赛事品牌影响力。加快九华国际康养候鸟基地、陌上星空、润昇康养园等项目建设，启动健康制造产业园建设，创成省级医养结合示范区。做好消费促进工作，引导网络消费、定制消费、体验消费等新模式新业态加快成长。推进百大物流等供应链物流项目建设，大力发展会展、工业设计、中介咨询等生产性服务业。

加快现代农业建设。大力培育绿色有机农产品加工业，加快3000吨黄精制品、高山蔬菜加工、金银花深加工等3个亿元以上项目建设，谋划引进茶叶、黄精等区域性特色产业和硒产业亿元以上项目5个，新增规模农产品加工企业10家以上。实施农产品区域公用品牌工程，借鉴“丽水山耕”模式，组建实体化的农业投资发展公司，力争用3-5年时间打造成池州名特优农产品“代言人”。推进国家森林生态标志产品建设工程，开展5个森标产品基地标准化示范建设。加快发展农业新业态，新增农业物联网企业10家，休闲农业综合收入34亿元以上，农村产品网络销售额18亿元以上。稳定粮食综合生产能力，发展稻鱼、稻虾共养10万亩以上。稳定生猪和家禽生产，强化重大动植物疫病防控。

**（三）突出补短板提品质，着力推动城乡一体化发展**

提高城市规划建设水平。全面实施主体功能区规划，加快打造美丽长江经济带池州段，深入推进全国绿化模范城市创建。编制实施城市设计总则、导则。加快三大功能区组团规划建设，南部天堂湖新区加快整体开发和功能配套，北部滨江区域推进岸线生态修复、文化遗址保护和业态谋划布局，东部产城融合创新区加快完善商业、科创、文体、教育等功能。建成牧之路互通立交工程，开工建设综合客运枢纽站。推进“城市双修”试点，加快湿地森林公园建设，新改建燃气供水管线80公里。建成平天湖三站、城防调度指挥系统，新改建排水管渠69.5公里。推广海绵城市建设理念，每个县区完成1-2个示范项目。促进房地产市场平稳健康发展。推进文明县城创建、无违建县区创建，提高县城吸引力和容纳力。支持创建1-2个省级县域特色产业集群（基地）。

全面实施交通建设大会战。建成池州长江公路大桥，加快池黄高铁、G3W德上高速池州段、九华山机场扩建项目建设。建成青阳至五溪“生态大道”，加快3条国省干线一级公路建设，开工S358青阳至红石等3条国省干线改建工程。启动江口轮渡北岸码头迁建。加快秋浦河航道整治工程建设，基本完成青通河航道整治。建成长九码头一期、江口港区三期，池州港跻身亿吨大港。完成池州长江公铁大桥工可编制，推进池州动车存车场、合肥—池州城际、池州—九江城际、宣城—石台—东至高速等项目前期工作。

强化城市精细化管理。完善数字化城市管理平台，健全监督评价机制。深入推进创建全国文明城市“十大整治”，启动美丽街区创建。更加注重解决物业“服务差、收费难”问题，落实行业监管和街道属地责任，推动已建成小区普遍成立业主委员会，促进物业服务按效付费，建立双向约束信用惩戒机制。更加注重解决城市交通陋习，打好机动车乱停乱放、电动车任性行驶、行人闯红灯三个专项整治持久战，加强停车场、停车泊位建设管理，让城市不再“添堵”。更加注重解决农贸市场脏乱差顽疾，抓好主城区农贸市场标准化改造和整治提升，理顺管理体制，让农贸市场回归公益属性。建设公交首末站，优化园区公交线路班次，方便市民出行。

打造文明宜居美好家园。学习浙江“千万工程”经验，深入推进农村人居环境整治三年行动，建成全部乡镇政府驻地和106个中心村污水处理设施，农村改厕2.3万户，农村生活垃圾处理率达到65%以上。强化村庄整治，复垦整理土地5500亩，启动2019年省级中心村建设。建成“四好农村路”扩面延伸1386公里。开工16座小型水库除险加固、6处中小河流治理等工程。加快9个大中型电网工程建设。推进乡村“四支人才”队伍建设。深入开展乡村文明行动。

**（四）突出抓重点建机制，着力打好三大攻坚战**

防范化解重大风险。健全以政府债券为主体的举债融资体制，妥善化解政府隐性债务风险。加强PPP项目合同、行业、资产管理，完善绩效评价、按效付费机制，支持各县区在污水处理、医疗、养老等领域扩大政府和社会资本合作。完善中小微企业信用体系，强化守信联合激励和失信联合惩戒，依法打击恶意逃废金融债务等行为。持续整治互联网金融，坚决遏制非法集资，推动不良贷款化解处置，加大政策性融资担保机构债务追偿、规范整顿力度，守住不发生系统性金融风险的底线。

打赢脱贫攻坚关键战役。深入推进“十大工程”，再减少贫困人口1万人。将新增扶贫资金、项目、举措向革命老区和深度贫困地区倾斜，加大“双基”建设力度，确保石台县如期高质量摘帽。大力推进“四带一自”“三有一网”“三业一岗”等扶贫模式，加强易地扶贫搬迁产业配套和就业安置，抓好扶贫车间建设。统筹推进非贫困村贫困户脱贫攻坚。大力实施扶贫扶志工程。深化结对帮扶和定点扶贫，全面开展“四送四通”直通车扶贫行动。

持续深化污染防治。抓好中央、省环保督察和“回头看”反馈问题整改，确保改彻底、改到位。巩固蓝天保卫战成果，建成大气质量网格化监测监管平台，严格落实“五控”措施，开展柴油车尾气专项治理、餐饮油烟提标整治行动，确保PM_2.5_平均浓度达到省考核要求。深入实施碧水攻坚战，加强集中式饮用水源地保护和备用水源地建设，完成城市及县城生活污水处理厂提标改造，消除县城建成区黑臭水体，实现入河排污口监督性监测全覆盖，确保国控、省控考核断面水质全面稳定达标。扎实推进净土持久战，大力实施土壤污染防治行动，在主城区全面推广生活垃圾分类，建成运营生活垃圾焚烧发电项目，严厉打击非法转移、倾倒固体废物和危险废物行为。巩固“六治行动”制度性成果，开展河湖“清四乱”专项行动，保持打击非法采砂高压态势。落实能耗、水资源消耗、建设用地总量和强度“双控”制度。

**（五）突出抓改革促开放，着力激发全社会创新创业活力**

加快重点领域改革步伐。坚持向改革要动力，推动各项改革走深走实。以管资本为主加强国有资产监管，积极稳妥推进混合所有制改革，推行职业经理人制度试点。加快市属四大集团公司转型发展，提升资本运作、项目运营和市场盈利能力。全面实施预算绩效管理，加大财政资金统筹整合力度。完成经营类事业单位转企改制。深化农村综合改革，有序实施农村土地“三权分置”制度，基本完成农村集体产权制度改革，全市40%以上的村完成“三变”改革。推深做实河湖长制、林长制。深化统计管理体制改革，做好第四次全国经济普查工作。

推动更高水平开放合作。与长三角、京津冀、珠三角等区域政府、开发园区、大院大所、产业联盟建立战略合作关系，积极融入长三角区域一体化，重点在铁路大通道、产业合作示范区、重大产业项目、旅游后花园建设等方面谋求突破。用活用好促进外资系列政策，实际利用外资3.9亿美元以上。加强与长三角城市群大通关建设协作，推进海关特殊监管区域扩能升级，新增进出口实绩企业15家，跨境电商贸易额增长10%以上。加快推进九华山机场开通临时直航包机。

开展“营商环境提升年”活动。以自我革命的勇气，加快营造稳定公平透明可预期的一流营商环境。全面实施市场准入负面清单制度，继续大力精简市级审批事项、办事流程，省内其他城市取消的都一律取消，保留事项的办理时限一律压到更短。深化“证照分离”改革，推行“双随机”跨部门联合抽查。推进政务服务“一网一门一次”改革再深化再升级，启用新政务服务大厅，实行“一窗受理、集成服务”。强化“四送一服”双千工程精准服务，开展产业扶持政策兑现、签约未落户项目问题排查、在建项目阻点“三个专项治理”，坚决做到守信践诺。完善政策执行方式，在环保、安全监管等领域执法过程中避免简单化、“一刀切”。建立招标重点项目随机抽查制度，严厉打击围标、串标行为。

加快社会治理创新。坚持发展新时代“枫桥经验”，深化“三社联动”等社区治理改革，扩大智慧社区试点。推进全民普法，扩大法律援助范围。加强预防和化解社会矛盾机制建设，依法及时解决群众合理诉求。支持工会、共青团、妇联等人民团体广泛参与社会管理和公共服务。促进民族团结进步。建立佛教商业化治理长效机制，依法加强宗教事务管理。

**（六）突出惠民生保民安，着力增强人民获得感幸福感**

加强就业和社会保障。扎实实施33项民生工程。常态化举办“2+N”就业招聘日活动，开展“接您回家”就业行动，鼓励和吸引池州籍在外人士返乡创业兴业。稳定和扩大全民参保覆盖面，完成城乡居民基本医疗保险制度整合，实行多元复合式医保支付方式。力争低保城乡统筹县区全覆盖。完善社会救助体系。推进公办养老机构改革试点，加强农村特困人员供养机构建设管理，县乡村三级社区居家养老服务中心覆盖率40%以上。加强农村留守儿童、妇女、老人关爱服务，抓好残疾人精准康复服务，支持红十字、慈善事业发展。

办好公平而有质量的教育。深入实施主城区基础教育提升计划，启动池州一中改造提升，新改建初中1所、小学4所，全市普惠性幼儿园覆盖率提高到80%以上。发挥职教集团作用，推进产教融合、校企合作。办好继续教育、特殊教育、老年教育，鼓励社会力量依法兴办教育。推进中小学教师“县管校聘”和“无校籍管理”改革。落实新时代教师职业行为十项准则，完善中小学教师待遇保障机制，让广大教师安心从教、热心从教。

推动卫生健康事业高质量发展。持续深化医药卫生体制综合改革，加快建立现代医院管理制度，支持组建紧密型医联体，推进分级诊疗、双向转诊。大力引进培育医疗卫生优秀紧缺人才，加强重点学科建设。深入实施医疗卫生服务能力三年行动提升计划，建成全民健康“一平台五中心”，抓好市儿童医院暨妇幼保健院、市医院外科综合楼等重点项目建设，乡镇卫生院、村卫生室标准化建设达标率分别达到85%、60%以上。推广社区卫生服务中心托管运营模式，提高家庭医生签约服务水平。创成省级卫生城市。推进计划生育转型发展。开展全民健康生活方式行动，创建健走示范城市。

加快建设文化名市。深入实施公民道德建设工程，建好用好池州好人馆。建成市委党校新校区，启用“一宫两中心”，进一步完善公共文化服务功能。加强文物保护利用和文化遗产保护传承，讲好“茶、傩、诗”等文化故事，实施革命遗址遗存保护利用工程，完成第二批历史建筑建档挂牌。组织庆祝建国70周年文艺创作展演。创建2个省级文化产业示范基地。实施全民科学素质行动，加强地方志、档案、广播电视、新闻出版、社科研究等工作。

全力维护安全稳定。以建设安徽最平安城市为抓手，加强平安池州建设。推进安全生产“铸安行动”常态化实效化，启动省级安全发展示范城市创建。抓好应急管理、气象、地震、地质灾害治理等工作，提升防灾减灾救灾综合能力。严防严控食品药品安全风险，严厉打击假冒伪劣商品向农村转移等违法行为。加快“雪亮工程”建设，完善立体化信息化社会治安防控体系，纵深推进扫黑除恶专项斗争。支持驻池部队建设，加强国家安全、国防教育、国防动员、民兵预备役和人民防空工作，启动市国防教育基地建设，推进军民深度融合发展，争创全国双拥模范城“五连冠”。

**三、全面加强政府自身建设**

人民对美好生活的向往，就是我们的奋斗目标。我们要把牢政治方向，牢固树立“四个意识”，坚决做到“两个维护”，不忘初心、牢记使命，努力创造经得起实践、人民、历史检验的实绩。

强化能力建设，切实做到思想解放、本领高强。扎实推进新一轮思想大解放，坚决破除狭隘封闭、因循守旧、不思进取、自甘落后，对标先进，找准短板，以思想破冰引领行动突围。加强学习型政府建设，大兴调查研究之风，在高质量发展的实践中弥补知识弱项、能力短板、经验盲区，不断提升政策运用、产业谋划、项目推动和群众工作的能力，集中精力谋划推进一批事关长远发展的重大课题、重大项目，把池州的机遇和潜力加快转化为改革发展的蓬勃态势。

强化作风建设，切实做到求真务实、担当作为。把抓落实作为政府工作的生命线，强化经济运行调度，拧紧明责、履责、督责、追责一体化的责任链条。持之以恒加强作风效能整顿，开展“万人评机关、评窗口、评科长”活动，对不问具体的“甩手掌柜”、不抓落实的“二传手”、不求质量的“差不多先生”坚决“零容忍”。完善激励约束、容错纠错机制，旗帜鲜明为担当干事者撑腰鼓劲、营造环境。

强化法治建设，切实做到依法行政、科学施政。牢固树立宪法意识、法治观念，严格依照法定权限、法定规则、法定程序行使权力、履行职责。以转变职能为核心，如期高质量完成市县政府机构改革任务，加快承担行政职能事业单位改革，深化综合行政执法改革。健全依法决策机制，做好地方立法工作。自觉接受人大法律和工作监督、政协民主监督，加强政府内部监督，主动接受社会和舆论监督，深入推进政务公开，让权力在阳光下运行。

强化廉政建设，切实做到清正清廉、纪律严明。认真贯彻中央八项规定精神和省、市委实施细则，狠刹“四风”特别是形式主义、官僚主义，严格规范督查检查考核，让基层腾出更多精力谋发展、抓落实。抓好巡视巡察和审计发现问题整改，从严管好公共资源、公共权力、公共资金。坚持勤俭办一切事业，压缩一般性支出，把有限的资金用在促发展、惠民生的刀刃上。认真履行政府系统从严治党主体责任和“一岗双责”，支持纪检监察机关依法依规履行职责，坚决向破坏发展环境、侵害群众利益的人和事亮剑，促进干部清正、政府清廉、政治清明。

各位代表！发展如逆水行舟，不进则退。让我们更加紧密地团结在以习近平同志为核心的党中央周围，在省委、省政府和市委的坚强领导下，解放思想、奋发进取，咬定目标、苦干实干，不断开创现代化绿色池州创新池州幸福池州建设新局面，以优异成绩庆祝新中国成立70周年！
